# Early results of totally endoscopic robotic aortic valve replacement: analysis of 4 cases

**DOI:** 10.1186/s13019-022-01899-3

**Published:** 2022-06-13

**Authors:** Jiaqi Sun, Ye Yuan, Yi Song, Yijie Hu, Xue Bai, Jing Chen, Qianjin Zhong

**Affiliations:** Department of Cardiovascular Surgery, Daping Hospital, Yuzhong District, Chongqing, China

**Keywords:** Robotic surgery, Aortic valve replacement, Minimally invasive surgery, Da Vinci SiTM system

## Abstract

**Objective:**

To evaluate the role of totally endoscopic robotic aortic valve replacement in cardiac surgery.

**Methods:**

Four cases of totally robotic aortic valve replacement (AVR) were conducted from December 2016 to July 2018. All operations were completed with the Da Vinci robot Si™ system (intuitive Surgical, Inc. Sunnyvale, C.A, USA). Patients were male, with a mean age of 42.8 ± 6.2 years (range 32–49).

**Results:**

AVR was completed with the Da Vinci Si™ system (intuitive Surgical, Inc. Sunnyvale, CA, USA). There was no mortality and no procedure-related morbidity. The mean cardiopulmonary bypass and mean cross-clamp time was 252 ± 13.6 min and 178.8 ± 17.1 min, respectively. The mean ICU time was 78.8 ± 27.1 h, and the mean hospital stay was 15 ± 3.5 d. During a mean follow-up of 3 years and 6 months, the patients returned to normal function, and no heart murmur was found. Compared with the operation, the body image score of the four patients increased after the operation, and the hospital anxiety and depression scale scores decreased, indicating that the patient's condition had been alleviated to a certain extent.

**Conclusion:**

Totally endoscopic robotic AVR is a feasible and viable choice for patients but requires further improvement for broader use.

**Supplementary Information:**

The online version contains supplementary material available at 10.1186/s13019-022-01899-3.

## Introduction

In recent years, with the aging of the population, the number of patients with aortic valve degenerative disease is also increasing. According to incomplete statistics, there are 200,000 operations for aortic valve disease in the world every year. The world's first robotic heart surgery was performed on May 7, 1998, by Alain Carpentier team using Da Vinci robot SiTM system prototype. Since then, robotic heart surgery has been applied in multiple heart diseases [1–4]. Robotic surgery has already proven itself to be of great value. Compared to conventional minimal surgery, robotic surgery can benefit from the magnified 3D surgical field, 7-degree-of-freedom mechanical arm, small size minimally invasive incision, an accurate and stable function of eliminating handshaking, and remote control, etc. [5]. In the experimental study of robot-assisted aortic valve replacement (AVR), Suri et al. reported their experience in using posterior seamless flap in cadaver model, and concluded that this method is equivalent to the traditional method and can be used clinically [6]. We conducted 163 cases of totally endoscopic robotic cardiac operations, including ventricular septal defect repair, atrial septal defect repair, mitral valve, and tricuspid valve replacement and repair, correction of anomalous pulmonary venous drainage, surgical treatment of partial atrioventricular canal defect, resection of cardiac tumor, resection of pericardial cyst, closure of coronary artery fistula, coronary artery bypass grafting, and hybrid procedure of percutaneous closure of a patent ductus arteriosus (PDA) and a totally robotic endoscopic vacuum sealing drainage (VSD) repair at our center since 2016 [7]. We conducted isolated AVR by standard median sternotomy for most patients, or by upper sternotomy or 3rd intercostal space for some cases. After accumulating experience of conventional and minimal AVR, we combined our experience with other types of robotic cardiac surgery and performed the totally endoscopic robotic AVR.

### Study design

This study is a retrospective and observational study of patients undergoing AVR with a full endoscopic robot.

### Patients

A total of 4 patients who underwent totally endoscopic robotic AVR were diagnosed and treated in our hospital from December 2016 to July 2018. All four patients had calcified aortic stenosis, and one of the patients had a concomitant atrial septal defect. Inclusion criteria are as follows: (1) age range 18–55 years; (2) slim body; (3) large aortic annulus; (4) wide sinus; (5) patients with aortic valve not severely calcified; (6) patients who meet other conditions for robotic surgery.

### Robot technology

**Fig. 1:**
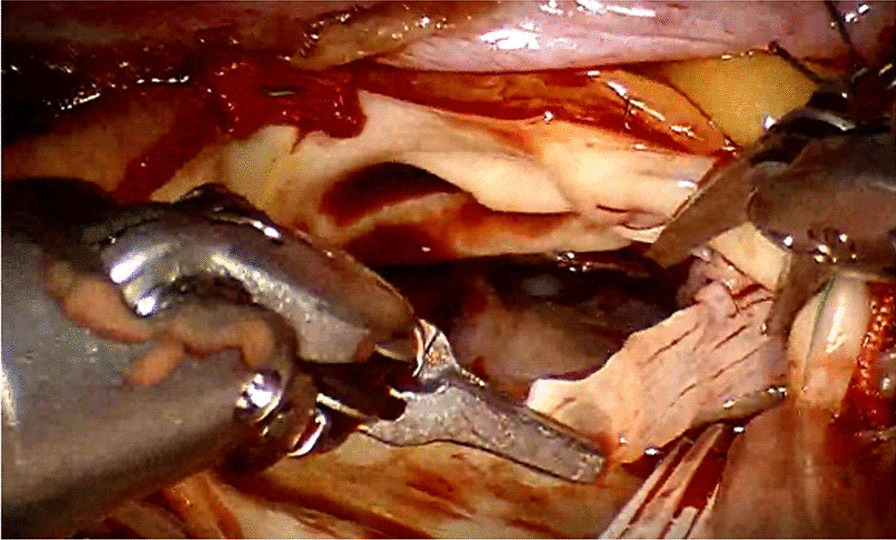
The Da Vinci robot Si™ system was used to excise the diseased valve

All procedures were completed with the Da Vinci robot Si™ system (Fig. 1). The working port and the endoscopic port were both placed at the 3/4th intercostal space (ICS). This working port with a Lap-protector (Huaren Pharmaceutical, Tsingtao, Shandong, China) was made instead of a mini-incision with a soft tissue retractor. It is provided for the assistant surgeon to co-operate with the head surgeon. But the assistant surgeon operated by watching the monitor indirectly. No maneuvers were performed under direct vision. The left and right arm ports were in the 2nd and 5th ICS. An occlusion port was placed in the 2nd/3rd ICS, and the hook port was placed at the 4/5 ICS (Additional file 1: Video 1).

### Surgical technique

**Fig. 2:**
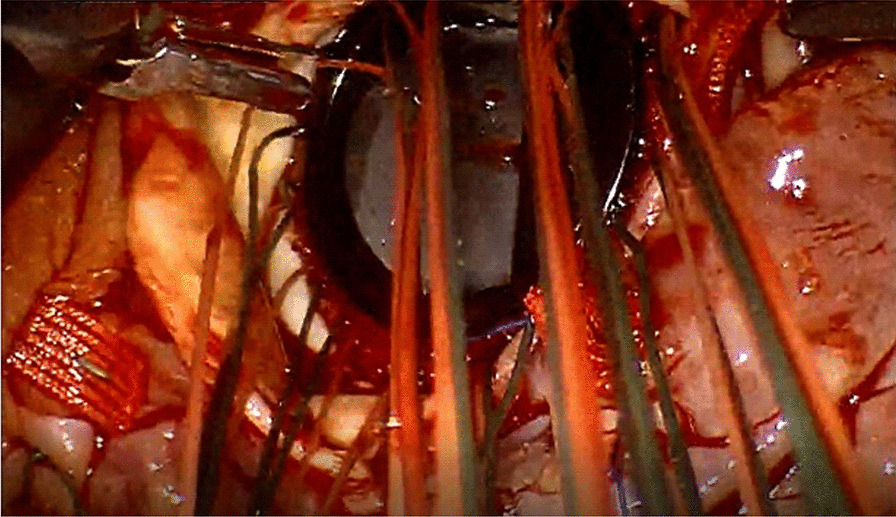
All the sutures were placed on the bileaflet mechanical prosthetic heart valve before it was implanted, and all knots were secured with a knot pusher

After being intubated with a double‐lumen endotracheal tube, the patient was positioned with the right chest elevated 30°. Cardiopulmonary bypass (CPB) was established through femoral arterial (18F), femoral venous (20F), and internal jugular venous (16F) cannulation. The patient cart was positioned on the left side of the patient, opposite the associate surgeon. An inverted T-shape incision was made on the pericardium. Two stay sutures were constructed next to the pericardial reentry on the left and right sides, fixed on the chest wall. Another two stay sutures were made on the lower and upper segments of the pericardium, fixed outside the working port. After the nasopharyngeal temperature dropped to 31 degrees, the ascending aorta cross-clamp was performed through the occlusion port, followed by delivering cold blood cardioplegia. After opening the right atrium, a 4–0 proline suture was used to sew a purse-bag around the tubular venous sinus, then, a reverse irrigation tube was inserted into the coronary sinus, and the purse-string was tightened for perfusion rather than using the water bag. The left ventricular venting was inserted through the right superior pulmonary vein. Carbon dioxide was insufflated continuously into the right pleural space for air displacement. The aorta was retracted with a hook, and the annulus was exposed by placing three sutures at each commissure. The temperature was maintained at 27℃ during CPB. Before the aorta clamp was removed, rewarming began until the anal temperature returned to 35℃ and the nasopharyngeal temperature returned to 36℃. No thermostatic blanket was put under the back of the patient. The aorta was incised with an oblique incision, lower on the noncoronary sinus side. The diseased valve was excised, and 15 stitches of 2/0 polypropylene sutures (Johnson & Johnson, Inc. New Brunswick. N.J, U.S.A) were placed under the annulus, five stitches for each phase. All the sutures were placed on the regent bileaflet mechanical prosthetic heart valve (Size 19 and 21, St. Jude Medical, Inc. East St. Paul, Minnesota, U.S.A.) before it was implanted and knots were secured with a knot pusher (Fig. 2). The incision on the ascending artery was closed with a polytetrafluoroethylene running suture (CV4, W.L. Gore & Associates, Inc. Newark. Delaware, USA). No evidence of retained air was found. The CPB group managed the body temperature. Finally, cardiopulmonary bypass was weaned and chest tubes were inserted. The total operative time of skin to skin was 431.3 ± 37.1 min. No conversions, no revision for bleeding, wound infection, and renal failure occurred. 1–2U erythrocytes and 1–2U plasma were transfused in our patients.

### Body image

The body image score measures a person's perception and gratification with their own body and explores the person's behavior toward the appearance of their body. The Body Image Questionnaire is a validated Likert-type instrument consisting of 40 items with 10 subscales assessing multiple aspects of body image (e.g., weight, facial features, muscle tone/definition, physical strength, overall appearance) [8]. A score of < 135 points is considered abnormal, denoting an altered perception of body image.

### Hospital anxiety and depression scale (HADS)

In general, the HADS-A was developed as a brief measure of generalized symptoms of anxiety and fear. The purpose of the HADS was to screen for clinically significant anxiety and depressive symptoms in medically ill patients. The scale consists of 14 items divided into two subscales: the HADS-A (Anxiety, seven questions) and HADS-D (Depression, seven questions) [9]. A 4-point Likert scale was provided for every item, and the highest score obtained for each subscale was 21. Scores of 0–7 in each subscale were evaluated as “normal,” while ≥ 8 indicated morbidity.

## Results

The characteristics of 4 patients who underwent totally endoscopic robotic AVR were diagnosed and treated in our hospital from December 2016 to July 2018 are shown in Table 1.

**Table 1:** The characteristics of 4 patients who underwent totally endoscopic robotic AVR were diagnosed and treated in our hospital

Patient	Gender	Age (y)	Height (cm)	Weight (kg)	Body surface area (m^2^)
1	Male	44	162	71	1.74
2	Male	46	175	72	1.88
3	Male	32	159	55	1.56
4	Male	49	171	72	1.84
Average		42.75 ± 6.18	165.33 ± 6.49	67.5 ± 7.23	1.75 ± 0.12

After totally endoscopic robotic aortic valve replacement, the effect indexes are shown in Table 2. The mean cardiopulmonary bypass and mean cross-clamp time were 271.75 ± 20.10 min and 183.75 ± 15.20 min, respectively. The mean ICU time and mean mechanical ventilation time were 83.25 ± 23.54 h and 21.25 ± 10.50 h. The mean value of volume of drainage was 452.5 ± 141.66 ml (Table 2). Patient-reported outcome measures of the patients are shown in Table 3. Compared with the operation, the body image score of the four patients increased after the operation, and the hospital anxiety and depression scale scores decreased, indicating that the patient's condition had been alleviated to a certain extent.

**Table 2:** Effect indexes of patients after complete endoscopic robotic aortic valve replacement

Patient	Cardiopulmonary bypass time (min)	Cross-clamp time (min)	ICU time (h)	Mechanical ventilation time (h)	Volume of drainage (ml)
1	280	202	55	13	620
2	260	193	119	39	530
3	247	162	72	14	240
4	300	178	87	19	420
Average	271.75 ± 20.10	183.75 ± 15.20	83.25 ± 23.54	21.25 ± 10.50	452.5 ± 141.66

**Table 3:** Patient-reported outcome measures of the patients

Patient	Body image score (40–200)		HAD-anxiety		HAD-depression	
	Pre-Op	Post-Op	Pre-Op	Post-Op	Pre-Op	Post-Op
1	150	158	**6**	**5**	**5**	**3**
2	143	147	**5**	**4**	**6**	**3**
3	159	163	**5**	**3**	**4**	**4**
4	140	147	**6**	**4**	**5**	**4**

HAD, Hospital Anxiety and Depression

The intraoperative and postoperative transesophageal echocardiograms demonstrated that the aortic valve works well. The patients returned to normal cardiac function without any complications. During a mean follow-up of 3 years and 6 months, all patients returned to normal function, and no heart murmur was found.

## Discussion

Arterial valve disease is one of the most common adult heart diseases in China. There are many causes of the disease. Whether it is a congenital valvular malformation, rheumatic disease, degenerative valve disease or infective endocarditis, it will reduce the function of left ventricular outflow tract, cause hemodynamic changes and increase the load of left and right ventricles [10]. In addition, with the progress of the disease, patients gradually have obvious symptoms of heart failure and other complications, which seriously threaten the life and quality of life of patients [11–13]. In the past 20 years, minimally invasive cardiac surgery (MICs) has developed by leaps and bounds and has been widely used all over the world [14]. It is different from the classic cardiac surgery in the past 50 years (such as median thoracotomy, longitudinal splitting of sternum for extracardiac surgery or heart valve surgery with cardiopulmonary bypass) [15]. AVR can be completed through a conventional median sternotomy with low morbidity, relatively low cost, and an excellent long-term result. However, the long incision in the median of the chest makes patients reluctant to undergo the conventional AVR [16, 17]. Also, it may result in bleeding, infection, and extended hospital stay. Furthermore, the incision leaves an unpleasant scar, which could be a source of persistent psychological problems and dissatisfaction. Because there is a very close relationship between body image and self-esteem, Michal and others insist that scars caused by heart surgery may have a considerable impact on the patient's body image and several aspects of daily life [18]. Our results tend to support this view. However, small incisions or various special surgical instruments are used for some heart surgery. One of the technical cores is to reduce or reduce the physical and mental trauma of the patient by reducing the surgical incision on the premise of ensuring the safety of the patient's operation. Previous studies have reported the feasibility of some cardiac procedures using robotic surgical systems, including mitral valve repair or replacement, coronary artery bypass grafting, repair of atrial septal defects (ASD) and partial anomalous pulmonary venous connections (PAPVC) [19–21]. With less blood loss, fewer incisions, and shorter hospital admissions, robotic cardiac surgery has proved to be a progressive technique [22].

Although reports have proved the effectiveness and safety of robot assisted cardiac surgery, it has not become the standard of care for the treatment of heart diseases. Through the efforts in our institution, we successfully operated four cases of totally endoscopic robotic AVR. Although reports have proved the effectiveness and safety of robot assisted cardiac surgery, it has not become the standard of care for the treatment of heart diseases. We used bicaval cannulation and an opening of the right atrium for direct insertion of a coronary sinus catheter in our surgery and the reasons are as follows: first, the opening of part of the coronary vein in the coronary sinus may be blocked after the water bag is used, causing the cold perfusion to not evenly enter the coronary vein. Second, considering that the blocking time may be longer, a more reliable myocardial protection solution perfusion is required to achieve a good myocardial protection effect.

The disadvantages of totally endoscopic robotic AVR include: (1) the lack of complete hook results in some trouble when exposing the aortic valve; (2) too much time was spent on knotting; (3) It is difficult for assistants to co-operate; (4) cold blood cardioplegia retrograde was delivered through the coronary sinus; (5) putting sutures in the annulus requires consummate surgical technique. Above all, we consider that the procedure of totally endoscopic robotic AVR is complex and time-consuming. Totally endoscopic surgery in other fields has shown improved quality of life but with longer clamping and CPB times during the learning curve [23, 24]. Though the clamping and CPB times were a little long for the complex operation and knotting in our cases, we believe they could be shorter with more practice. Furthermore, we believe that practice on animal tissues such as pig hearts before going clinically could help decrease risks. Also, inadequate instruments may result in difficulties and inconvenience, which may lead to more time consumption. Therefore, in the future, we think it is necessary to research some dedicated instruments for different robotic cardiac surgery and realize the specialization of surgical instruments.

## Conclusion

Totally endoscopic robotic aortic valve replacement is beneficial to patients with heart disease. We believe that it may become a feasible and viable choice for patients.

## Supplementary Information

**Additional file 1.** **Video 1.** The essential steps of totally endoscopic robotic aortic valve replacement.

## Data Availability

The data will be available on request to the corresponding author.

## References

[1] Sepehripour AH, et al. Robotics in cardiac surgery. A R Coll Surgeons Engl. 2018. 10.1308/rcsann.supp2.22 PMC6216752 30179050

[2] Peterson ED, Shroyer ALW (2016). Trends in robotic-assisted coronary artery bypass grafts: a study of the society of thoracic surgeons adult cardiac surgery database, 2006 to 2012. Ann Thorac Surg.

[3] Nifong LW, Chu VF, Bailey BM (2003). Robotic mitral valve repair: experience with the da Vinci system. Ann Thorac Surg.

[4] Mack MJ. Minimally invasive and robotic surgery. JAMA J Am Med Assoc. 2001. 10.1001/jama.285.5.568 11176860

[5] Cohn LH, Adams DH, Couper GS (1997). Minimally invasive cardiac valve surgery improves patient satisfaction while reducing costs of cardiac valve replacement and repair. Ann Surg.

[6] Cosgrove DM, Sabik JF, Navia JL (1998). Minimally invasive valve operations. Ann Thorac Surg.

[7] Hu Y, Deng J, Zhao S, et al. Left thorax approach to repair doubly committed juxta‐arterial ventricular septal defect with Da Vinci robotic system. J Cardiac Surg. 2019. 10.1111/jocs.14036 30981213

[8] Steinemann DC, Raptis DA, Lurje G (2011). Cosmesis and body image after single-port laparoscopic or conventional laparoscopic cholecystectomy: a multicenter double blinded randomised controlled trial (SPOCC-trial). BMC Surg.

[9] Herrmann C (1997). International experiences with the hospital anxiety and depression scale-a review of validation data and clinical results. J Psychosom Res.

[10] Grossi EA, LaPietra A, Ribakove GH (2001). Minimally invasive versus sternotomy approaches for mitral reconstruction: comparison of intermediate-term results. J Thorac Cardiovasc Surg.

[11] Yamada T, Ochiai R, Takeda J (2003). Comparison of early postoperative quality of life in minimally invasive versus conventional valve surgery. J Anesth.

[12] Walther T, Falk V, Metz S (1999). Pain and quality of life after minimally invasive versus conventional cardiac surgery. Ann Thorac Surg.

[13] Felger JE, Nifong LW, Chitwood WR (2001). The evolution and early experience with robot-assisted mitral valve surgery. Curr Surg.

[14] Vola M, Fuzellier J-F (2014). First human totally endoscopic aortic valve replacement: an early report. J Thoracic Cardiovasc Surg.

[15] Folliguet TA, Vanhuyse F, Konstantinos Z, et al. Early experience with robotic aortic valve replacement. Eur J Cardiothor Surg. 2005. 10.1016/j.ejcts.2005.03.021 15982601

[16] Balkhy HH, Ctp L, Kitahara H (2018). Robot-assisted aortic valve surgery: state of the art and challenges for the future. Rev Int J Med Robot.

[17] Fukutomi M, Hokken T, Wong I, et al. Prophylactic permanent pacemaker strategy in patients with right bundle branch block undergoing transcatheter aortic valve replacement. Catheter Cardiovasc Interv. 2021. 10.1002/ccd.29914 34390167

[18] Iyigün T, Kaya M, Gülbeyaz SO (2017). Patient body image, self-esteem, and cosmetic results of minimally invasive robotic cardiac surgery. Int J Surg.

[19] Onan B, Kadirogullari E, Guler S, Kahraman Z (2018). Robotic-assisted removal of an Amplatzer atrial septal occluder device for residual shunting, closure of septal defect and simultaneous tricuspid annuloplasty. J Robot Surg.

[20] Onan B, Aydin U, Basgoze S, Bakir I (2016). Totally endoscopic robotic repair of coronary sinus atrial septal defect. Interact Cardiovasc Thorac Surg.

[21] Pirelli L, Kliger CA, Patel NC (2017). Minimally invasive robotically assisted repair of partial anomalous venous connection. Innovations (Phila).

[22] Vola M, Fuzellier JF, Gerbay A, Campisi S (2016). First in human totally endoscopic Perceval valve implantation. Ann Thorac Surg.

[23] Bonatti J, Wallner S, Winkler B, Grabenwöger M (2020). Robotic totally endoscopic coronary artery bypass grafting: current status and future prospects. Expert Rev Med Devices.

[24] Nisivaco S, Henry M, Ward RP, Balkhy HH (2019). Totally endoscopic robotic-assisted excision of right ventricular papillary fibroelastoma. J Robot Surg.

